# Perceived Norms and Vaccination against COVID-19 among the General Adult Population in Germany: Results of a Nationally Representative Survey

**DOI:** 10.3390/vaccines11040800

**Published:** 2023-04-04

**Authors:** André Hajek, Benedikt Kretzler, Boris Orth, Ursula Von Rüden, Hans-Helmut König

**Affiliations:** 1 Department of Health Economics and Health Services Research, University Medical Center Hamburg-Eppendorf, Hamburg Center for Health Economics, 20246 Hamburg, Germany; 2 Federal Centre for Health Education (BZgA), Referat Q3–Evaluation, Methods, Research Data, 50825 Cologne, Germany

**Keywords:** COVID-19, SARS-CoV-2, vaccination, GP, norms

## Abstract

Objective: to examine whether perceived norms are associated with vaccination against COVID-19 (also stratified by age group). Study design: nationally representative survey. Methods: Data were taken from a sample of the general adult population (*n* = 3829, 16 to 94 years). Data collection took place from early July to early August 2021, and 3 different groups (1: not yet vaccinated and no intention to vaccinate against COVID-19; 2: not yet, but intended to vaccinate against COVID-19; 3: yes, at least one vaccination against COVID-19) were distinguished in the analyses. Data were adjusted for several sociodemographic and health-related factors. Perceived norms served as key independent variables (1: number of important friends and relatives who would like me to get vaccinated; 2: number of important friends and relatives who already have been vaccinated or still want to do so; 3: how your general practitioner (GP) thinks about Corona vaccination). Results: Multiple logistic regression showed that, in particular, the number of important friends/relatives who would like an individual to get vaccinated is associated with the actual COVID-19 vaccination status among individuals aged 16 to 59 years. Interestingly, all 3 indicators for perceived norms are associated with the likelihood of COVID-19 vaccination status among individuals aged 60 years and over. Conclusions: Our study adds to the understanding of the association between perceived norms and COVID-19 vaccination status. This highlights potential pathways to increase vaccination rates to further combat the later stages of the pandemic.

## 1. Introduction

Since early 2020, the Corona pandemic has dominated discussion in nearly every area of life and not only in Germany. Since the end of 2020/beginning of 2021, vaccinations against COVID-19 that are effective [1,2,3]—at least in the sense that they protect against severe disease progression—can be administered. The determinants of vaccination against COVID-19 have been examined in a variety of studies [4,5]. However, most of the existing studies focused on rather ‘classical’ determinants (such as sociodemographic factors) or psychological factors such as beliefs in the effectiveness or side effects of the vaccines [4,5].

To date, the role of *perceived norms* for vaccination against COVID-19 is quite poorly understood. According to the Theory of Planned Behavior [6,7], the intention to perform a certain behavior contributes to the actual behavior and is influenced by perceived norms [8]. Varol et al. [8] defined perceived norms as “one’s perception that important others might (dis)approve of them for engaging in a behavior (injunctive norm) and one’s perception that others like themselves do (or do not) engage in a behavior (descriptive norm)” (page 2). We also follow this definition. Varol et al. showed that perceived norms determine COVID-19 vaccine intention—based on data from an online survey among students from Maastricht University [8]. To this end, they quantified perceived norms using three items (each on a seven-point Likert scale from disagree to fully agree) [8]. Their exact wording was as follows [8]: “I think that most people like me will get the COVID-19 vaccination”; “I think that my doctor/health care provider wants me to get the COVID-19 vaccination”; and “I think that most people who are important to me want me to get the COVID-19 vaccination” (page 3), which is comparable to our study.

Similar findings were also obtained in recent studies based on convenience or student samples [9,10,11]. Thus, the aim of this study was to investigate the association between perceived norms and COVID-19 vaccination status based on a *nationally representative sample* (also stratified by age group).

We assume that there may be differences in the association between perceived norms and vaccination status depending on age, as perceived norms might be of particular importance for the decision to vaccinate among individuals aged 60 years and over. More precisely, we assume that older individuals—in comparison to younger individuals—may pay particular attention to the opinion of their general practitioner (GP), with whom they have a regular, long-standing, trusting, and respectful relationship [12], not least because it has been shown that they are often satisfied with the care provided by the GP [13].

Knowledge about the link between perceived norms and the vaccination against COVID-19 may, among other aspects, assist in increasing the rate of the first (recommended for all adult individuals) and the second booster vaccination (recommended for individuals aged from 60 years on and individuals at increased risk of severe disease progression, such as individuals with cancer). In Germany, for example, the first booster vaccination rate was 51.9% among individuals aged 18 to 59 years and 77.6% among individuals aged 60 years and over [14] (mid-October 2022 [14]). The second booster vaccination rate is only 13% among individuals aged 60 years and over in Germany in mid-October 2022 [14]. A potential conclusion may be that it is important to encourage GPs to recommend the second booster vaccination rate among older adults, which in turn may contribute to maintaining health among older individuals. It can also help keep hospitalization rates low and, in doing so, can help to avoid overburdening the health care system.

## 2. Methods

### 2.1. Sample

In 2021, three representative surveys (called “Kommunikation der Corona-Schutz-impfung in Deutschland” (CoSiD; regarding representativeness, please see [15]) of the general population (i.e., the German-speaking population aged 16 and older) and particular subgroups (such as individuals aged 66 and over as well as medical professionals with patient contact) were conducted by the Federal Centre for Health Education (“Bundeszentrale für gesundheitliche Aufklärung”, BZgA). COVID-19 immunization (in terms of knowledge level, attitudes, and behavior) was the main subject of the CoSiD study.

Sampling, data collection, and weighting were carried out by the market and opinion research institute “INFO GmbH Markt- und Meinungsforschung” on behalf of the BZgA. The most recent ADM master sample (https://www.adm-ev.de/en/services/the-adm-sampling-system/, accessed on 29 March 2023) served as the basis for sampling selection, covering landline as well as mobile telephone numbers. In addition, a quota-based subsample was obtained from an actively recruited online access panel of Norstat Deutschland GmbH. A pretest was conducted. The first representative survey’s field time fell between 9 July and 5 August 2021. About three-quarters (73%) of the interviews were conducted via Computer-Assisted Telephone Interviewing (CATI). Additionally, about one-quarter (27%) of the interviews were conducted via Computer-Assisted Web Interviewing (CAWI). The average interview lasted 28 min (CATI) and 24 min (CAWI).

In the CATI sample, the age group of 66–85-year-olds was disproportionately increased. In sum, the response rate of the telephone survey was 14%. The contact persons’ (41%) or targets’ (43%) refusals were the most common causes of dropouts. Based on the most recent Federal Statistical Office (i.e., microcensus) data, sample weighting was used to ensure representativeness. Household size, age, gender, education, and federal state were taken into account. In the total analytical sample, *n* = 3829 individuals were included (which means that only 3 individuals were dropped in total due to missing data).

### 2.2. Outcomes

First, individuals were asked whether they had already received a vaccination against COVID-19 (options: yes, once; yes, twice; not yet). Individuals who had not yet received a vaccination against COVID-19 were asked about their intentions to vaccinate against COVID-19 (options: definitely vaccinate; rather vaccinate; undecided; rather not vaccinate; definitely not vaccinate).

Based on these two questions, we formed three groups:

- Group 1: not yet vaccinated and no intention to vaccinate against COVID-19 (including individuals without a vaccination who responded with “rather not vaccinate” or “definitely not vaccinate” to the question regarding the intention to vaccinate against COVID-19).
- Group 2: not yet, but intended to vaccinate against COVID-19 (including individuals without a vaccination who responded with “undecided”, “rather vaccinate”, or “definitely vaccinate” to the question regarding the intention to vaccinate against COVID-19).
- Group 3: yes, at least one vaccination against COVID-19 (including individuals who already received one or two vaccinations against COVID-19).

### 2.3. Independent Variables

Three indicators for perceived norms served as key independent variables. In the first question, individuals were asked: “What is your personal environment’s opinion of the Corona vaccination? How many individuals in your family and acquaintances whose opinion is important to you would like you to get vaccinated?” (Options: nobody; a few; about half; most; all).

Moreover, individuals were asked: “how many friends and relatives whose opinion is important to you have already been vaccinated or still want to do so” (options: nobody; a few; about half; most; all).

Subsequently, individuals were asked: “Can you tell me what your general practitioner’s position is on Corona vaccination?” (Options: My general practitioner… recommends the Corona vaccination to me; … advises me against the Corona vaccination; … does not give me any recommendation regarding the Corona vaccination; I have not talked to my general practitioner about the vaccination; I do not have a general practitioner).

### 2.4. Covariates

Based on former research and theoretical considerations [4,5], covariates were selected for regression analysis: sex (men; women); age (in years); being in a relationship/partnership (including marriage) (yes; no); highest school or university degree (No school-leaving qualification (school finished without graduation), Certificate of Secondary Education/Elementary School Diploma/POS 8th/9th grade or diploma after no more than 7 years of school attendance, General Certificate of Secondary Education/POS 10th grade or equivalent qualification, Abitur/Specialized university entrance qualification/Advanced technical college entrance qualification, Degree from a University of Applied Sciences/Degree from a College or University); labor force participation (employed; retired; other (including: exclusively househusband/housewife; unemployed; student; in training; studying at university/technical college; on parental leave/maternity leave)), and birth country (Germany; born abroad). Moreover, self-rated health (from 1 = very good to 5 = very bad), and a count score for chronic conditions (from 0 to 6; Asthma and Chronic Obstructive Pulmonary Disease (COPD); Cardiac or circulatory disease(s); Liver and kidney disease(s); Neurological disease(s) (e.g., multiple sclerosis); Metabolic disease(s) (e.g., diabetes); Congenital or acquired immunodeficiency(s)) were included as covariates in a regression analysis.

### 2.5. Statistical Analysis

First, sample characteristics were determined. Then, multinomial logistic regressions (base outcome: not yet vaccinated and no intention to vaccinate against COVID-19) were conducted to investigate the association between perceived norms and vaccination against COVID-19. This was adjusted for various sociodemographic and health-related factors in a regression analysis, as described in the above section.

Statistical significance was defined as *p*-value of <0.05. Stata 16.1 was used for statistical analyses (Stata Corp., College Station, TX, USA).

## 3. Results

### 3.1. Sample Characteristics

In our analytical weighted sample, the average age was 50.4 years (95% CI: 49.5–51.4 years, 16 to 94 years), and 51.1% (95% CI: 48.7–53.4%) of the individuals were female. In terms of vaccination status, most individuals (80.8% of the individuals, 95% CI: 78.8–82.7%) belonged to the third group (i.e., had at least 1 vaccination against COVID-19). Moreover, the most frequently chosen answer category for our first two key independent variables reflecting perceived norms was ‘most’, closely followed by ‘all’. Just over half of the respondents (53.0%, 95% CI: 50.6–66.3%) received a recommendation for the Corona vaccination by their GP. Further details are given in Table 1.

### 3.2. Regression Analysis

The results of multinomial logistic regressions (also stratified by age group) are shown in Table 2 (base outcome was group 1). Relative risk ratios (RRR) are displayed in Table 2 (with 95% CI in parentheses). For reasons of clarity and readability, we focus on the results comparing group 1 (i.e., individuals who are not yet vaccinated and have no intention to vaccinate against COVID-19) and group 3 (i.e., individuals who have at least one vaccination against COVID-19). Furthermore, for reasons of readability, concrete figures only are shown in Table 2.

The likelihood of being vaccinated (i.e., group 3) compared to being unvaccinated and without an intention to vaccinate (i.e., group 1) is lower when only *a few* or *none* (compared to about half) of an individual’s own friends and relatives (whose opinion is important to him or her) would like him or her to get vaccinated. This applies to the total sample as well as for individuals aged 16 to 59 and individuals aged 60 years and over.

Conversely, the likelihood of being vaccinated compared to being unvaccinated and without an intention to vaccinate is higher when *most* or *all* (compared to about half) of an individual’s friends and relatives (whose opinion is important to him or her) would like him or her to get vaccinated. This applies to the total sample as well as for individuals aged 16 to 59 and individuals aged 60 years and over (with one exception among individuals aged 60 years and over: ‘most’ compared to ‘about half’).

In comparison to these results, there is a significant association between the number of friends and relatives (whose opinion is important to the individuals) who already have been vaccinated or intend to do so and the status of being vaccinated among the total sample and individuals aged 60 years and over, but not among individuals aged 16 to 59 years.

Additionally, the likelihood of being vaccinated compared to being unvaccinated and without an intention to vaccinate is much lower when general practitioners either did not give any recommendation regarding the Corona vaccination, or did not talk about the vaccination with the patient, or when individuals did not have a general practitioner (compared to general practitioners who recommended the vaccination against COVID-19). This applies to the total sample as well as for individuals aged 16 to 59 and mainly for individuals aged 60 years and over. It is worth noting that the likelihood of being vaccinated compared to being unvaccinated and without an intention to vaccinate is much lower when the GP advised against the COVID-19 vaccination (compared to the case when the GP recommended the vaccination against COVID-19) among individuals aged 60 years and over (but not among the total sample nor among individuals aged 16 to 59 years).

It is worth emphasizing that when *all* friends and relatives (whose opinion is important to an individual) would like the individual to get vaccinated, the individual had a much higher likelihood of being vaccinated against COVID-19 compared to when *about half* of such friends and relatives would like an individual to get vaccinated (across all models).

## 4. Discussion

Based on a large representative sample of the general adult population in Germany, our aim was to examine the association between perceived norms and COVID-19 vaccination (also stratified by age group). Our current study markedly enriches our understanding about such a link often mainly based on selective samples [9,10,11] and thus rarely based on nationally representative samples (e.g., Japan [16]).

Among individuals aged 16 to 59 years, the number of important friends/relatives who would like an individual to get vaccinated is particularly important to the actual COVID-19 vaccination status in our study. Factors such as social pressure (to be able to actively participate in joint activities, for example) or fears about health consequences could be decisive here [6]. Moreover, in this age bracket, the active recommendation of the GP (compared to a missing recommendation of the GP, the lack of discussions about the vaccination between the GP and the patient, or the general lack of a GP) is important for the COVID-19 vaccination status in our study. We conclude that an active recommendation could reduce potential uncertainties regarding the safety or efficacy of vaccination among individuals in this age group.

Interestingly, among individuals aged 60 years and over, all 3 indicators of perceived norms are associated with the likelihood of the COVID-19 vaccination status in our study. It is also possible that older individuals perceive a particular social pressure or a responsibility to society as a whole. However, further research is needed to explore the mechanisms of these associations among older adults.

It is also worth noting that active advice against the Corona vaccination by a GP can heavily influence the actual vaccination status in this age bracket (in contrast to individuals aged 16 to 59 years). We assume that older adults pay extra attention to the advice of GPs considering the fact that they may often have a regular, established, and trust-based relationship with their GP [12]. Furthermore, the recommendation against vaccination must always be seen in the context of an individual’s medical history (i.e., there could be medically justifiable reasons against vaccination in individual cases), particularly among older adults.

We would like to note some strengths and limitations of our study. It should be highlighted that we used data from a carefully planned nationally representative survey. Our study also included the oldest old individuals—an important (but heavily neglected) group during the pandemic due to the potential health consequences of an infection with SARS-CoV-2. Moreover, it was able to distinguish between groups with different vaccination statuses: vaccinated people, as well as unvaccinated individuals with and without an intention to get vaccinated. Furthermore, different perceived norms (such as the position of GPs on vaccination against COVID-19) were used as key independent variables. However, it should be acknowledged that our study had a cross-sectional design. Additionally, a low response rate is a clear limitation of our study. However, low response rates are rather common in telephone surveys conducted in Germany and do not necessarily affect the representativeness [17]. Moreover, sampling weights were applied to account for study design and non-response.

## 5. Conclusions

This study adds to our understanding of the association between perceived norms and vaccination against COVID-19. For example, our study stresses the importance of GP recommendations among older adults. This highlights potential ways to increase vaccination rates to further combat the later stages of the pandemic. For example, explicit recommendations by GPs for vaccination could prove useful among older adults in Germany, e.g., to increase the meager current second booster vaccination rate.

## Figures and Tables

**Table 1:** Sample characteristics (weighted analytical sample, *n* = 3829).

Variables	Mean and 95% CI in Parentheses/ Frequency and 95% CI in Parentheses (in %)
Age: Mean and 95% CI in parentheses	50.4 (49.5–51.4)
Sex: Frequency and 95% CI in parentheses	
- Men	48.9 (46.6–51.3)
- Women	51.1 (48.7–53.4)
Highest school or university degree: Frequency and 95% CI in parentheses	
- No school-leaving qualification (school finished without graduation)/Certificate of Secondary Education/Elementary School Diploma/POS 8th/9th grade or diploma after no more than 7 years of school attendance	18.1 (16.4–20.0)
- General Certificate of Secondary Education/POS 10th grade or equivalent qualification	45.7 (43.3–38.0)
- Abitur/Specialized university entrance qualification/Advanced technical college entrance qualification	20.8 (19.0–22.8)
- Degree from a University of Applied Sciences/Degree from a College or University	15.4 (13.9–17.0)
Employment status: Frequency and 95% CI in parentheses	
- Employed	51.3 (49.0–53.7)
- Retired	30.8 (28.8–32.9)
- Other: not employed	17.9 (16.0–19.9)
Being in a partnership/relationship: Frequency and 95% CI in parentheses	
- Yes	65.9 (63.6.68.2)
- No	34.1 (31.9–36.4)
Birth country: Frequency and 95% CI in parentheses	
- Germany	92.4 (90.9–93.7)
- Born abroad	7.6 (6.3–9.1)
Self-rated health (from 1 = very good to 5 = very bad): Mean and 95% CI in parentheses	2.1 (2.1–2.2)
Count score for chronic conditions (from 0 to 6): Mean and 95% CI in parentheses	0.4 (0.3–0.4)
Number of individuals in my own family and acquaintances (whose opinion is important to me) who would like me to get vaccinated: Frequency and 95% CI in parentheses	
- Nobody	4.2 (3.3–5.3)
- A few	8.3 (7.1–9.7)
- About half	11.9 (10.3–13.6)
- Most	38.4 (36.2–40.7)
- All	37.3 (35.0–39.6)
Number of individuals in my own family and acquaintances (whose opinion is important to me) who are already vaccinated or would like to be vaccinated: Frequency and 95% CI in parentheses	
- Nobody	3.0 (2.2–4.1)
- A few	9.4 (8.0–10.9)
- About half	11.3 (9.9–12.9)
- Most	43.0 (40.7–45.3)
- All	33.4 (31.2–35.6)
General practitioner’s position on Corona vaccination: Frequency and 95% CI in parentheses	
- Recommends the Corona vaccination to me	53.0 (50.6–66.3)
- Advises me against the Corona vaccination	2.2 (1.5–3.0)
- Does not give me any recommendation regarding the Corona vaccination	4.5 (3.6–5.6)
- I have not talked to my general practitioner about the vaccination	35.9 (33.6–38.2)
- I do not have a general practitioner	4.5 (3.6–5.6)
COVID-19 vaccination: Frequency and 95% CI in parentheses	
- Group 1: not yet vaccinated and no intention to vaccinate against COVID-19	9.9 (8.6–11.5)
- Group 2: not yet, but intended to vaccinate against COVID-19	9.3 (7.9–10.8)
- Group 3: yes, at least one vaccination against COVID-19	80.8 (78.8–82.7)

Notes: The category “other” (employment status) includes: exclusively househusband/housewife, unemployed, student, in training, studying at university/technical college, on parental leave/maternity leave.

**Table 2:** Determinants of vaccination against COVID-19. Results of multinomial logistic regression.

Independent Variables	Group 2—Among Individuals of All Ages	Group 3— Among Individuals of All Ages	Group 2— Among Individuals Aged 16 to 59 Years	Group 3— Among Individuals Aged 16 to 59 Years	Group 2— Among Individuals Aged 60 Years +	Group 3—Among Individuals Aged 60 Years +
Age (in years)	0.99	1.00	0.99	0.99	1.02	1.04
	(0.97–1.01)	(0.98–1.02)	(0.96–1.01)	(0.97–1.02)	(0.95–1.10)	(0.97–1.11)
Sex						
- Men	1	1	1	1	1	1
- Women	1.22	1.21	1.00	1.22	3.20 *	0.86
	(0.74–2.00)	(0.78–1.90)	(0.57–1.77)	(0.72–2.06)	(1.04–9.82)	(0.40–1.84)
Employment status						
- Employed	1	1	1	1	1	1
- Retired	1.25	2.45 *	0.43	0.58	11.65 **	11.33 ***
	(0.52–3.03)	(1.09–5.51)	(0.10–1.91)	(0.14–2.35)	(2.15–63.03)	(3.11–41.23)
- Other	1.09	0.54 *	0.95	0.47 *	278.00 **	33.83 *
	(0.59–1.99)	(0.30–0.97)	(0.51–1.80)	(0.25–0.87)	(8.10–9537.84)	(1.29–890.74)
Being in a partnership/relationship—						
- Yes	1	1	1	1	1	1
- No	1.65 +	1.62 *	1.27	1.43	4.79 *	2.55
	(0.97–2.80)	(1.01–2.58)	(0.71–2.28)	(0.84–2.44)	(1.43–16.02)	(0.81–8.06)
Education						
- No school-leaving qualification (school finished without graduation)/Certificate of Secondary Education/Elementary School Diploma/POS 8th/9th grade or diploma after no more than 7 years of school attendance)	1	1	1	1	1	1
- General Certificate of Secondary Education/POS 10th grade or equivalent qualification	1.16	0.92	1.57	1.26	0.44	0.48
	(0.57–2.36)	(0.50–1.70)	(0.66–3.72)	(0.58–2.76)	(0.15–1.27)	(0.18–1.28)
- Abitur/Specialized university entrance qualification/Advanced technical college entrance qualification	1.79	1.89	2.45+	2.49 *	0.17+	0.49
	(0.76–4.23)	(0.87–4.09)	(0.92–6.48)	(1.01–6.15)	(0.03–1.05)	(0.14–1.80)
- Degree from a University of Applied Sciences/Degree from a College or University	0.83	1.51	1.14	1.94	0.56	1.40
	(0.31–2.21)	(0.68–3.36)	(0.36–3.57)	(0.73–5.18)	(0.13–2.44)	(0.39–5.06)
Birth country						
- Germany	1	1	1	1	1	1
- Born abroad	0.93	0.72	1.02	0.69	0.01 *	0.59
	(0.41–2.10)	(0.32–1.64)	(0.41–2.55)	(0.27–1.77)	(0.00–0.41)	(0.16–2.17)
Self-rated health (from 1 = very good to 5 = very bad)	1.26	0.98	1.38 +	1.03	0.83	0.79
	(0.92–1.73)	(0.71–1.35)	(0.96–1.98)	(0.72–1.48)	(0.31–2.22)	(0.32–1.99)
Count score for chronic conditions (from 0 to 6)	0.47 **	0.84	0.57 +	1.04	0.45	0.76
	(0.29–0.74)	(0.58–1.22)	(0.31–1.03)	(0.62–1.72)	(0.17–1.22)	(0.32–1.78)
Number of individuals in my own family and acquaintances (whose opinion is important to me) who would like me to get vaccinated						
- nobody	0.48	0.13 ***	0.41	0.17 ***	0.45	0.05 ***
	(0.18–1.26)	(0.05–0.32)	(0.13–1.32)	(0.06–0.47)	(0.04–4.60)	(0.01–0.28)
- a few	0.80	0.29 ***	0.84	0.34 **	0.81	0.16 **
	(0.38–1.65)	(0.16–0.52)	(0.38–1.84)	(0.17–0.66)	(0.13–5.05)	(0.05–0.51)
- about half	1	1	1	1	1	1
- most	4.31 **	3.71 ***	4.24 **	3.87 **	3.20	2.44
	(1.71–10.85)	(1.84–7.48)	(1.54–11.70)	(1.71–8.72)	(0.59–17.30)	(0.68–8.77)
- all	45.37 ***	33.91 ***	94.40 ***	82.87 ***	21.78 **	4.82 *
	(8.91–230.95)	(7.97–144.29)	(7.40–1203.45)	(7.41–927.51)	(3.42–138.82)	(1.12–20.84)
Number of individuals in my own family and acquaintances (whose opinion is important to me) who are already vaccinated or would like to be vaccinated						
- nobody	0.50	0.28 *	0.32 +	0.36	0.89	0.25
	(0.17–1.48)	(0.09–0.94)	(0.09–1.20)	(0.10–1.28)	(0.10–8.29)	(0.05–1.31)
- a few	0.54 +	0.51 *	0.60	0.54 +	0.21 +	0.27 *
	(0.27–1.07)	(0.28–0.95)	(0.28–1.24)	(0.27–1.07)	(0.04–1.04)	(0.09–0.87)
- about half	1	1	1	1	1	1
- most	0.53	2.13 *	0.57	1.98 +	0.47	5.08 **
	(0.22–1.28)	(1.09–4.19)	(0.22–1.47)	(0.92–4.27)	(0.11–2.04)	(1.69–15.30)
- all	0.23 *	1.68	0.23 +	1.50	0.18 +	5.11 *
	(0.06–0.88)	(0.56–5.07)	(0.05–1.18)	(0.37–6.10)	(0.03–1.27)	(1.08–24.18)
General practitioner’s position on Corona vaccination:						
- recommends the Corona vaccination to me	1	1	1	1	1	1
- advises me against the Corona vaccination	0.59	0.46	0.67	0.48	0.03 **	0.04 ***
	(0.18–1.89)	(0.15–1.39)	(0.20–2.25)	(0.15–1.57)	(0.00–0.26)	(0.01–0.17)
- Does not give me any recommendation regarding the Corona vaccination	0.51	0.31 *	0.52	0.29 *	0.15	0.20
	(0.18–1.42)	(0.12–0.85)	(0.17–1.58)	(0.10–0.85)	(0.01–2.46)	(0.03–1.65)
- I have not talked to my general practitioner about the vaccination	0.50 *	0.21 ***	0.51 +	0.20 ***	0.65	0.30 **
	(0.26–0.95)	(0.13–0.36)	(0.25–1.02)	(0.11–0.38)	(0.24–1.78)	(0.13–0.67)
- I do not have a general practitioner	0.66	0.18 ***	0.81	0.19 ***	0.01 **	0.11 **
	(0.26–1.67)	(0.07–0.43)	(0.31–2.13)	(0.07–0.49)	(0.00–0.35)	(0.02–0.53)
Constant	1.12	7.34 **	0.99	6.73 *	0.05	0.62
	(0.25–5.04)	(1.98–27.17)	(0.17–5.78)	(1.46–30.98)	(0.00–9.66)	(0.01–42.79)
Pseudo R^2^	0.34	0.34	0.31	0.31	0.50	0.50
Observations	3829	3829	1388	1388	2441	2441

Relative risk ratios (RRR) are reported; 95% CI in parentheses; *** *p* < 0.001, ** *p* < 0.01, * *p* < 0.05, + *p* < 0.10. Base outcome: Group 1: not yet vaccinated and no intention to vaccinate against COVID-19 (including individuals without a vaccination who responded with “rather not vaccinate” or “definitely not vaccinate” to the question regarding the intention to vaccinate against COVID-19). Group 2: not yet, but intended to vaccinate against COVID-19 (including individuals without a vaccination who responded with “undecided”, “rather vaccinate”, or “definitely vaccinate” to the question regarding the intention to vaccinate against COVID-19. Group 3: yes, at least one vaccination against COVID-19 (including individuals who already received one or two vaccinations against COVID-19).

## Data Availability

The datasets analyzed during the current study are not publicly available due to ethical restrictions.

## References

[1] Lopez Bernal J., Andrews N., Gower C., Gallagher E., Simmons R., Thelwall S., Stowe J., Tessier E., Groves N., Dabrera G. (2021). Effectiveness of COVID-19 vaccines against the B. 1.617. 2 (Delta) variant. N. Engl. J. Med..

[2] Andrews N., Tessier E., Stowe J., Gower C., Kirsebom F., Simmons R., Gallagher E., Thelwall S., Groves N., Dabrera G. (2022). Duration of protection against mild and severe disease by COVID-19 vaccines. N. Engl. J. Med..

[3] Andrews N., Stowe J., Kirsebom F., Toffa S., Rickeard T., Gallagher E., Gower C., Kall M., Groves N., O’Connell A.M. (2022). COVID-19 vaccine effectiveness against the Omicron (B. 1.1. 529) variant. N. Engl. J. Med..

[4] Kafadar A.H., Tekeli G.G., Jones K.A., Stephan B., Dening T. (2022). Determinants for COVID-19 vaccine hesitancy in the general population: A systematic review of reviews. J. Public Health.

[5] Wang Y., Liu Y. (2021). Multilevel determinants of COVID-19 vaccination hesitancy in the United States: A rapid systematic review. Prev. Med. Rep..

[6] Ajzen I. (2015). The theory of planned behaviour is alive and well, and not ready to retire: A commentary on Sniehotta, Presseau, and Araújo-Soares. Health Psychol. Rev..

[7] Xiao X., Wong R.M. (2020). Vaccine hesitancy and perceived behavioral control: A meta-analysis. Vaccine.

[8] Varol T., Schneider F., Mesters I., Ruiter R.A.C., Kok G., Ten Hoor G.A. (2022). Facilitating Informed Decision Making: Determinants of University Students&rsquo; COVID-19 Vaccine Uptake. Vaccines.

[9] Lachance-Grzela M., Charbonneau A., Dubé A., Jbilou J., Richard J. (2022). Parents and caregivers’ willingness to vaccinate their children against COVID-19. Can. J. Behav. Sci./Rev. Can. Des Sci. Comport..

[10] Guidry J.P., Laestadius L.I., Vraga E.K., Miller C.A., Perrin P.B., Burton C.W., Ryan M., Fuemmeler B.F., Carlyle K.E. (2021). Willingness to get the COVID-19 vaccine with and without emergency use authorization. Am. J. Infect. Control.

[11] Mo P.K., Luo S., Wang S., Zhao J., Zhang G., Li L., Li L., Xie L., Lau J.T. (2021). Intention to receive the COVID-19 vaccination in China: Application of the diffusion of innovations theory and the moderating role of openness to experience. Vaccines.

[12] Keller J., Slee J.A. (2001). Older people’s perceptions of their general practitioner. Australas. J. Ageing.

[13] Greene M.G., Adelman R.D., Friedmann E., Charon R. (1994). Older patient satisfaction with communication during an initial medical encounter. Soc. Sci. Med..

[14] Robert-Koch-Institut (2022). Tabelle mit den Gemeldeten Impfungen nach Bundesländern und Impfquoten nach Altersgruppen (17.10.2022, Tabelle Wird Montags bis Freitags Aktualisiert). https://www.rki.de/DE/Content/InfAZ/N/Neuartiges_Coronavirus/Daten/Impfquotenmonitoring.html.

[15] Bosle C., Orth B., Reibling N., Merkel C., Muschalik C., von Rüden U. (2022). Gesundheitsinformationsverhalten und Gesundheitskompetenzen zur COVID-19-Schutzimpfung von Menschen in Deutschland—Befunde der CoSiD-Studie. Bundesgesundheitsblatt-Gesundh.-Gesundh..

[16] Nomura S., Eguchi A., Yoneoka D., Kawashima T., Tanoue Y., Murakami M., Sakamoto H., Maruyama-Sakurai K., Gilmour S., Shi S. (2021). Reasons for being unsure or unwilling regarding intention to take COVID-19 vaccine among Japanese people: A large cross-sectional national survey. Lancet Reg. Health-West. Pac..

[17] Hajek A., Lehnert T., Wegener A., Riedel-Heller S.G., König H.-H. (2017). Who should take care of me? Preferences of old age individuals for characteristics of professional long-term caregivers: An observational cross-sectional study. BMC Res. Notes.

